# In-Hospital outcomes in acute coronary syndrome patients with concomitant severe chronic kidney disease undergoing percutaneous coronary intervention

**DOI:** 10.12669/pjms.35.2.276

**Published:** 2019

**Authors:** Saadia Sattar, Naseer Ahmed, Zohaib Akhter, Saba Aijaz, Shakir Lakhani, Rehan Malik, Asad Pathan

**Affiliations:** 1 *Saadia Sattar, M.Sc Epi-Bio. Research Consultant, Department of Clinical Research, Cardiology, Tabba Heart Institute, Karachi, Pakistan*; 2 *Naseer Ahmed, MBBS Cardiology Fellow, Department of Cardiology, Tabba Heart Institute, Karachi, Pakistan*; 3 *Zohaib Akhter, M.Sc Epi-Bio. Research Consultant, Department of Clinical Research, Cardiology, Tabba Heart Institute, Karachi, Pakistan*; 4 *Saba Aijaz, FCPS. Consultant Cardiologist, Cardiology, Tabba Heart Institute, Karachi, Pakistan*; 5 *Shakir Lakhani, FCPS. Consultant Cardiologist, Cardiology, Tabba Heart Institute, Karachi, Pakistan*; 6 *Rehan Malik, Research Officer, Department of Cardiology, Tabba Heart Institute, Karachi, Pakistan*; 7 *Asad Pathan, FACC. Consultant Cardiologist, Cardiology, Tabba Heart Institute, Karachi, Pakistan*

**Keywords:** Acute coronary syndrome, Renal Insufficiency, Percutaneous Coronary Intervention

## Abstract

**Objective:**

To determine in-hospital mortality and major adverse cardiac events (MACE) in acute coronary syndrome (AMI) patients with underlying severe chronic kidney disease (CKD) undergoing percutaneous coronary intervention (PCI).

**Methods:**

We conducted a retrospective cohort study from June’2013-December’2017 at Tabba Heart Institute, Karachi. Data was drawn from institutes’ database modeled after US National Cardiovascular data CathPCI registry. All AMI (STEMI: ST-elevation myocardial infarction and NSTEMI: non-ST-elevation myocardial infarction) patients undergoing PCI with creatinine clearance <30ml/min or ESRD on hemodialysis were included in the study.

**Results:**

During 54 months study period, 160 severe CKD patients underwent PCI. Mean age was 62.9±12.2 years. Men were 61.9%, hypertensive (81.3%) and diabetic (63.8%). Excluding dialysis patients, Creatinine clearance was 21.1±6.6ml/min/1.73m^2^. STEMI were 46.9% and 61.9% were Killip I. Mean SYNTAX score was 16.6±7.3. MACE occurred in 32.5% patients, of which 6(11.5%) had new hemodialysis and mortality: 17.5% were deceased. MACE predictor were cardiogenic shock (OR: 2.81, 95%CI: 1.17-6.74) and prior heart failure (OR: 6.84, 95%CI: 1.39-33.74), Predictor of mortality was cardiogenic shock or cardiac arrest (OR: 7.90, 95%CI: 2.95-21.17).

**Conclusion:**

Severe CKD patients undergoing PCI for AMI have drastically poor outcomes therefore individualization and patient-centric care management is mandatory.

## INTRODUCTION

Chronic kidney disease (CKD) is a rapidly increasing public health concern and the mortality of severe CKD and end stage renal disease (ESRD) patients is very high as compared to the general population.^1^ Cardiovascular disease is the most common cause of death in these patients in the past, accounting for half of the mortality with an annual 18-20% death rate.^2^-^6^ CKD patients presenting with acute myocardial infarction (AMI) are a particularly high-risk group with data from the late 1990’s showing 41% mortality at one year and up to 71% at five years.^7^

Patients with CKD also fare unfavorably during PCI with increased risk of bleeding complications, peri-procedural myocardial infarction, stent thrombosis and increased mortality.^8^ Renal function deterioration after PCI contributes further to a poorer prognosis in patients with preexisting CKD with a reported 6.8% in-hospital mortality^9^ and >70% mortality over the period of two years.^10^ In the absence of outcome data of the additive benefits of revascularization in patients with AMI and severe CKD, clinicians may adopt a risk aversion approach and not recommend cardiac catheterization or revascularization.^11^

Clinical trials of cardiovascular therapies, in general exclude patients with severe CKD, and clinicians have to rely on retrospective, observational registries and meta-analysis to make patient care decisions.^11^ All these studies are published from North America, Europe or Japan; there is very limited data available from south Asia. Patients of these areas may have a different risk profile from Western, Caucasian and Oriental population and based on this we sought to determine the in-hospital outcomes of AMI patients with severe CKD undergoing PCI.

## METHODS

We conducted a retrospective cohort study from June 2013 through December 2017 at Tabba heart Institute Karachi, Pakistan. Data was retrieved from institute’s patient database modeled according to standard United States National Cardiovascular Data Registry (NCDR) Cath-PCI registry.^12^ The registry collects data on patient and characteristics, clinical presentation, treatments, and outcomes associated with coronary angiography and/or PCIs. There is a comprehensive data quality program, including both data quality report specifications for data capture and transmission and an auditing program. The data collected are exported in a standard format. The complete definitions of all variables were prospectively defined by a committee of the American College of Cardiology (ACC) and are available at the ACC NCDR Website.

For this study, we identified all AMI (STEMI: ST-elevation myocardial infarction and NSTEMI: non-ST-elevation myocardial infarction) patients of age ≥18 years undergoing PCI having creatinine clearance <30ml/min or on hemodialysis. We excluded patients undergoing multiple PCI procedures during a single hospitalization. Approval from Tabba Heart Institute Ethics Review Committee was obtained for the conduct of the study. Informed consent was taken from all patients at the time of admission for research purposed while maintaining confidentiality.

### Study outcomes

It included in-hospital mortality and in-hospital major adverse cardiac events (MACE). In-hospital mortality was defined as mortality due to any cause post PCI. In-hospital MACE included heart failure, cerebrovascular accident/stroke, bleeding event within 72 hours, myocardial infarction and new need of hemodialysis.

### Operational definition

Estimated glomerular filtration rate (eGFR; ml/min/1.73m^2^) using initial serum creatinine of the patient was calculated by modification of diet in renal disease (MDRD) formula: eGFR = 186 x Serum Cr-1.154 x age-0.203 x 1.212 (if patient is black) x 0.742 (if female).^13^ Dialysis patients are considered as end stage renal disease (ESRD) and their creatinine clearance data was not included in the creatinine clearance calculation in our study population. All AMI patients (STEMI/NSTEMI) were included. STEMI or their equivalents were characterized by the presence of: a. symptoms suggestive of acute coronary ischemia. b. ECG evidence of STEMI: New or presumed new ST-segment elevation or new left bundle branch block with the cut-off points: ≥0.2 mV in men or ≥0.15mV in women in leads V2-V3 and/or ≥0.1 mV in other leads or true posterior infarcts. Left bundle branch block (LBBB) refers to new or presumed new LBBB on the initial ECG. NSTEMIs were characterized by the presence of: a. symptoms suggestive of acute coronary ischemia. b. Cardiac biomarkers (Troponin I) exceeding the diagnostic criteria cutoffs of Tabba Heart Institute’s laboratory parameters with a clinical presentation which is consistent or suggestive of ischemia. c. ECG changes not meeting STEMI criteria. Heart failure was defined as unusual dyspnea on light exertion or recurrent dyspnea occurring in the supine position or fluid retention or the description of rales or jugular venous distension or pulmonary edema on physical exam; or pulmonary congestion on chest x-ray. Cerebrovascular accident was documented by a loss of neurological function caused by an ischemic or hemorrhagic event with residual symptoms lasting at least 24 hours after onset or leading to death. MI was defined as repeat symptoms of coronary ischemia with new ECG change. Biomarkers were not routinely checked after PCI and are only done in patients with suggestion of repeat ischemia. Bleeding event within 72 hours was defined as hemoglobin drop of >3 g/dL or transfusion of whole blood or packed red blood cells or procedural intervention/surgery at the bleeding site to reverse/stop or correct the bleeding (such as surgical closures/exploration of the arteriotomy site, balloon angioplasty to seal an arterial tear, endoscopy with cautery of a GI bleed). New hemodialysis; was defined as nonhemodialysis dependent patient requiring hemodialysis due to acute or worsening renal failure. Multi vessel disease was defined as significant blockage in more than one of the major coronary artery.

### Statistical Plan

Results are presented as mean ± SD for continuous variables and frequency with percentages for categorical variables. Continuous data were compared by means of Student’s *t* test; proportions by Chi-square test. Stepwise multivariable logistic model was developed for clinically relevant variables, to identify risk factors of in-hospital mortality and in-hospital MACE adjusting for CKD. Results are presented as odds ratios (ORs) with 95% CIs. p-value <0.05 was considered statistically significant.

## RESULTS

### Study population and demographic characteristics

During the 54 months study period, a total of 7133 patients underwent PCI of which 160 (2.2%) patients met the inclusion criteria of AMI presentation and underlying severe CKD. Patients already on hemodialysis were 13.1%. Among the study patients, mean age was 62.9±12.2 years, majority were men (61.9%) (Table-I). Hypertension was present in 81.3%, dyslipidemia in 35.0%, diabetes in 63.8% and 13.1% of the patients were smokers. Prior history of revascularization with either PCI or CABG was present in 23.1% of the patients. Initial Creatinine was mean of 3.4 ± 1.9 mg/dl and Creatinine clearance was 21.1 ± 6.6 ml/min/1.73m^2^.

**Table-I:** Demographic, clinical, angiographic and procedural characteristics.

Characteristics	In-hospital Event			p-value

	Total (n=160)	Alive (n=132)	Deceased (n=28)	
Demographic and clinical characteristics				
Age (years)*	62.9±12.2	62.9±11.7	63.3±14.6	NS
Male	99 (61.9)	78 (59.1)	21 (75.0)	0.12
Diabetes mellitus	102 (63.8)	84 (63.6)	18 (64.3)	NS
Insulin treatment	46 (46.0)	42 (51.2)	4 (22.2)	<0.001^#^
Hypertension	130 (81.3)	114 (86.4)	16 (57.1)	<0.001
Dyslipidemia	56 (35.0)	52 (39.4)	4 (14.3)	0.01^#^
Smokers	21 (13.1)	18 (13.6)	3 (10.7)	NS^#^
Prior revascularization	37 (23.1)	34 (25.8)	3 (10.7)	0.08
Prior known CAD	46 (28.7)	42 (31.8)	4 (14.3)	0.06^#^
Family history of premature CAD	28 (17.5)	24 (18.2)	4 (14.3)	0.NS^#^
Cardiac arrest at arrival	13 (8.1)	7 (5.3)	6 (21.4)	0.005^#^
In-hospital stay (days)*	5.5±3.7	5.8±3.6	4.2±3.8	0.02
Killip class on presentation				
I	99 (61.9)	88 (66.7)	11 (39.3)	<0.001^#^
II	10 (6.3)	8 (6.1)	2 (7.1)	
III	28 (17.5)	26 (19.7)	2 (7.1)	
IV	23 (14.4)	10 (7.6)	13 (46.4)	
Initial Creatinine (mg/dl)	3.4±1.9	3.4±2.1	3.0±0.8	0.27
eGFR (ml/min/m^2^)	21.1±6.6	20.8±6.7	22.7±5.9	0.18
Angiographic and Procedural Characteristics				
Pre PCI LVEF (%)*	40.6±10.6	41.5±10.4	34.2±9.9	0.005
Significant blockage				
Left main disease	5 (3.1)	5 (3.2)	1 (10.7)	NS^#^
Multi vessel disease	58 (43.9)	49 (37.1)	9 (32.1)	
Single vessel disease	97 (60.6)	79 (59.7)	18 (64.3)	
SYNTAX score*	16.6±7.3	16.0±7.4	21.7±4.7	0.07
SYNTAX score risk categories				
Low	45 (83.3)	41 (85.4)	4 (66.7)	NS
Intermediate	7 (12.9)	5 (10.4)	2 (33.3)	
High	2 (3.7)	2 (4.2)	0 (0.0)	
Total (available)	54 (100)	48 (100)	6 (100)	
PCI treated vessel 1	108 (67.5)	87 (65.9)	21 (75.0)	NS
On hemodialysis	21 (13.3)	18 (13.6)	3 (10.7)	NS
PCI Indication				
STEMI	75 (46.9)	54 (40.9)	21 (75.0)	
-within 24 hours	60 (37.5)	43 (32.6)	17 (60.7)	0.001^$^
NSTEMI	85 (53.1)	78 (59.1)	7 (25.0)	
Contrast volume (ml)	111.3±78.1	107.8±74.2	128.4±94.4	0.23
Shock at start of PCI	17 (10.6)	6 (4.6)	11 (39.3)	<0.001
Post procedure TIMI 3 flow	156 (97.5)	132 (100)	24 (85.7)	0.001
Acute kidney injury	57 (35.6)	46 (34.9)	11 (39.3)	NS

* mean±SD

# Fisher’s exact test.

$ p-values between STEMI and NSTEMI.

### Clinical presentation

A total of 46.9% patients’ presented with STEMI and 53.1% patients with NSTEMI. Majority of the patients presented with Killip I (61.9%). Cardiac arrest before PCI occurred 8.1% and 14% had cardiogenic shock on presentation

### Angiographic and procedural characteristics

Mean ejection fraction was 40.6%±10.6 and one third of the patients had ejection fraction <35%. Severe left main artery or multi vessel disease was present in 47%.SYNTAX score was available for 54 patients [Alive: 48 (36.4%), dead: 6 (21.4%)]. Mean SYNTAX score was 16.6±7.3 (Alive: 16.0±7.4, dead: 21.7±4.7).Single vessel PCI was done in 67.5% and post procedure TIMI 3 flow was achieved in 97.5% of the patients.

### Post procedural event

**Fig.1:**
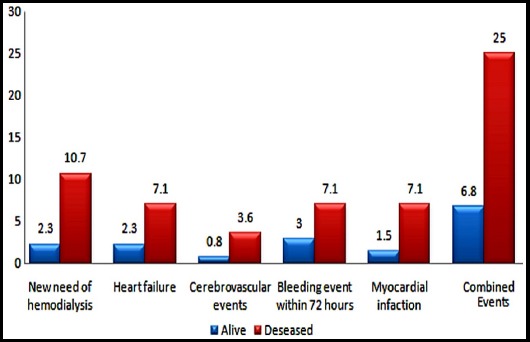
Post procedural events.

In-hospital mortality was 17.5% (STEMI: 28.0%, NSTEMI: 8.2%). Deceased patients were more likely to have cardiac arrest (21.4% vs. 5.3%), KILLIP IV on presentation (46.4% vs. 7.6%), slightly higher SYNTAX score (21.7±4.7 vs. 16.0±7.4), STEMI presentation (75.0% vs. 40.9%) and TIMI flow <3 (85.7% vs. 100%) (all p<0.05).Post procedure events were more likely to occur in deceased patients (Fig.1). A total of 52 (32.5%) patients had one of the major adverse cardiac events including death. Mean hospital stay was 5.5 ± 3.7 days.

### Predictors of In-hospital mortality and MACE

In multivariate analysis, MACE was more likely to occur in patients having cardiogenic shock (OR: 2.81, 95%CI: 1.17-6.74, p-value: 0.02) and prior heart failure (OR: 6.84, 95%CI: 1.39-33.74, p-value: 0.02) (Table-II). However, predictor of in-hospital mortality was cardiogenic shock or cardiac arrest (OR: 7.90, 95%CI: 2.95-21.17, p-value: <0.001).

**Table-II:** Predictors of in-hospital mortality and MACE (n=160).

	Multivariate models							

Variable	In-hospital mortality^*^				In-hospital MACE^#^			

	OR	p-value	95%CI		OR	p-value	95%CI	
	
			Lower	Upper			Lower	Upper
Cardiogenic shock or cardiac arrest	7.90	<0.001	2.95	21.17	2.81	0.02	1.17	6.74
Prior heart failure	-	-	-	-	6.84	0.02	1.39	33.74

* Gender, Insulin treatment, prior PCI, prior known CAD, cardiac arrest at arrival, in-hospital stay, Killip class, eGFR, multi vessel disease, PCI indication and TIMI 3 flow, AKI, shock at start of PCI were found insignificant in multivariable model

# Age, Gender, Insulin treatment, hypertension, dyslipidemia, prior PCI, prior known CAD, cardiac arrest at arrival, in-hospital stay, Killip class, PCI indication and TIMI 3, AKI flow were found insignificant in multivariable model

## DISCUSSION

In this study, we looked at the in-hospital outcomes in 160 patients with AMI and severe CKD who underwent PCI. We observed that these patients had multiple co-morbidities, frequent cardiac arrest and cardiogenic shock at presentation. They usually have a prolonged hospital stay, worsening of renal function in a third of patients, frequently complicated with adverse events and high in-patient mortality of 17.5%. Cardiogenic shock or prior heart failure predicted in-hospital adverse events.

Cardiovascular diseases are the leading cause of death in patients with severe CKD or ESRD.^14^ Renal dysfunction was shown in the HOPE trial to be a marker of increased cardiovascular risk and CKD is now considered a CHD equivalent.^15^ Majority of patients already have obstructive CHD at the time of initiation of dialysis and there is usually presence of multi-vessel disease with calcified, diffuse involvement.^16^ Patients with worsening degree of CKD have increasing cardiovascular events with a very high risk associated with ESRD.^17^ In addition to an increased burden of traditional cardiovascular risk factors, presence of non-traditional risk factors such as anemia and a superimposed amplified pro-inflammatory state mediate the enhanced coronary and systemic burden of atherosclerosis.^18^ An alteration in calcium and phosphate metabolism present in renal dysfunction is associated with intimal calcium deposition in CKD and medial calcification in the coronary arteries with ESRD.^19^ There is marked endothelial dysfunction, altered coronary perfusion and along with increased atherosclerosis, leads to increased cardiovascular mortality. Coronary revascularization with PCI or surgery is challenging in these patients due to the presence of multiple co-morbidities, diffusely diseased and calcified coronary arteries, aortic and systemic vascular disease; there is associated high morbidity and mortality.^20^

The mortality rate in our study for AMI patients with underlying severe CKD is very high at 17.5% and patients mostly had low SYNTAX score suggesting less complex coronary anatomy. At the same time in half of the patients, index hospitalization was for STEMI presentation; most of whom (31%) underwent emergency PCI within 24 hours of symptom onset. These patients have a higher risk of death and therefore an unfavorable influence on early survival. In a study from the late 1990’s, Herzog et al showed that in ESRD patients, the 2-year mortality after myocardial infarction was 74%.^21^ Results after revascularization in CKD patients are more limited. In the study from the European registry by Möckel et al, STEMI patients had a higher risk of death with an OR of 2.16.^22^ In the study by Shroff et al the patients with AMI and multi-vessel disease derived greater benefit from CABG as compared to PCI with DES; HR 0.92 for predicting mortality in DES versus CABG.^23^ However, patients presenting with acute STEMI should still be treated with PCI because of a shorter presentation to treatment time.

The overall rate of adverse events is very high in our study consistent with the high-risk population.^10^ Over 30% of patients had one of the adverse events; with death, heart failure, bleeding and dialysis being the more common. This highlights the importance of multiple risk reduction measures that are recommended in the guidelines and best practices: accurate measurement of creatinine clearance in all patients, adjustment of anticoagulants based on creatinine clearance and weight, careful assessment of volume status to avoid both volume depletion and volume overload in order to reduce heart failure and acute kidney injury, preference for radial artery access and minimizing iodinated contrast utilization with strict attention to safe contrast volume.^24^,^25^ The risk of contrast nephropathy can be further reduced by staging complex procedures and using intravascular ultrasound or optical coherence tomography guidance instead of fluoroscopy.^26^

We only found cardiogenic shock at presentation and a history of heart failure as predictors of in-hospital adverse events. This is probably explained by the small sample size. Previous studies have also found cardiogenic shock to be a strong predictor of in hospital death with 40-50% mortality in most studies.^27^ Most randomized trials excluded patients with severe CKD, and even in the observational registries, patients with the combination of severe CKD and AMI are poorly represented.^11^ This clearly represents a dilemma to the treating cardiologist, as generalizing data from trials where such patients were excluded can be misleading. These patients have a poor outcome with medical therapy which may imply use of more aggressive approaches. However, based on their advanced multiple co-morbidities, worse clinical presentation and complex medical problems, there is a much higher risk of adverse outcome; unfavorably affecting the risk benefit ratio of coronary revascularization procedures. This further stresses the importance of individualization and patient-centric care management.

### Limitations of the study

We acknowledge that this is the single-center experience represented through this data. This may limit the generalizability of conclusions. The sample size of the study was small which may lead to lack of significant predictors. There is also a possibility that predictors of in-hospital mortality or in-hospital mace (study outcomes) could reflect measured or unmeasured co-morbidities in patients with advance CKD.

## CONCLUSION

Cardiovascular diseases are the most frequent cause of mortality in patients with severe CKD and ESRD. Patients with severe CKD and associated AMI have historically a very poor outcome. These patients are not included in randomized trials and the observational registries have reported very limited or no data from South Asia.We observed that patients with severe CKD and AMI have multiple co-morbidities, frequent cardiac arrest and cardiogenic shock at presentation. They usually have a prolonged hospital stay, worsening of renal function in a third of patients, frequently complicated with adverse events and high inpatient mortality. There is need for individualization and patient-centric care management.

### Authors Contribution

**SS & ZA:** Conceived, designed and did statistical analysis & writing of manuscript.

**RM, NA, & SL:** Did data collection, data editing and editing of manuscript.

**AP & SA:** Did clinical interpretation of data, review and final approval of manuscript.
